# Discovery of cell active macrocyclic peptides with on-target inhibition of KRAS signaling†

**DOI:** 10.1039/d1sc05187c

**Published:** 2021-11-25

**Authors:** Shuhui Lim, Nicolas Boyer, Nicole Boo, Chunhui Huang, Gireedhar Venkatachalam, Yu-Chi Angela Juang, Michael Garrigou, Hung Yi Kristal Kaan, Ruchia Duggal, Khong Ming Peh, Ahmad Sadruddin, Pooja Gopal, Tsz Ying Yuen, Simon Ng, Srinivasaraghavan Kannan, Christopher J. Brown, Chandra S. Verma, Peter Orth, Andrea Peier, Lan Ge, Xiang Yu, Bhavana Bhatt, Feifei Chen, Erjia Wang, Nianyu Jason Li, Raymond J. Gonzales, Alexander Stoeck, Brian Henry, Tomi K. Sawyer, David P. Lane, Charles W. Johannes, Kaustav Biswas, Anthony W. Partridge

**Affiliations:** MSD International Singapore 138665 Singapore awpartridge@gmail.com; Merck & Co., Inc. Boston Massachusetts 02115 USA kaustav.biswas@merck.com; Agency for Science, Technology and Research (A*STAR) Singapore 138665 Singapore cwjohannes@gmail.com; Merck & Co., Inc. Kenilworth New Jersey 07033 USA; Merck & Co., Inc. West Point Pennsylvania 19486 USA

## Abstract

Macrocyclic peptides have the potential to address intracellular protein–protein interactions (PPIs) of high value therapeutic targets that have proven largely intractable to small molecules. Here, we report broadly applicable lessons for applying this modality to intracellular targets and specifically for advancing chemical matter to address KRAS, a protein that represents the most common oncogene in human lung, colorectal and pancreatic cancers yet is one of the most challenging targets in human disease. Specifically, we focused on KRpep-2d, an arginine-rich KRAS-binding peptide with a disulfide-mediated macrocyclic linkage and a protease-sensitive backbone. These latter redox and proteolytic labilities obviated cellular activity. Extensive structure–activity relationship studies involving macrocyclic linker replacement, stereochemical inversion, and backbone α-methylation, gave a peptide with on-target cellular activity. However, we uncovered an important generic insight – the arginine-dependent cell entry mechanism limited its therapeutic potential. In particular, we observed a strong correlation between net positive charge and histamine release in an *ex vivo* assay, thus making this series unsuitable for advancement due to the potentially fatal consequences of mast cell degranulation. This observation should signal to researchers that cationic-mediated cell entry – an approach that has yet to succeed in the clinic despite a long history of attempts – carries significant therapy-limiting safety liabilities. Nonetheless, the cell-active molecules identified here validate a unique inhibitory epitope on KRAS and thus provide valuable molecular templates for the development of therapeutics that are desperately needed to address KRAS-driven cancers – some of the most treatment-resistant human malignancies.

## Abstract 2

Targeting undruggable intracellular proteins with peptides: novel on-target macrocyclic peptide inhibitors of KRAS with broad inhibition of proliferation of multiple KRAS-dependent cancer cell lines.

## Graphical abstract

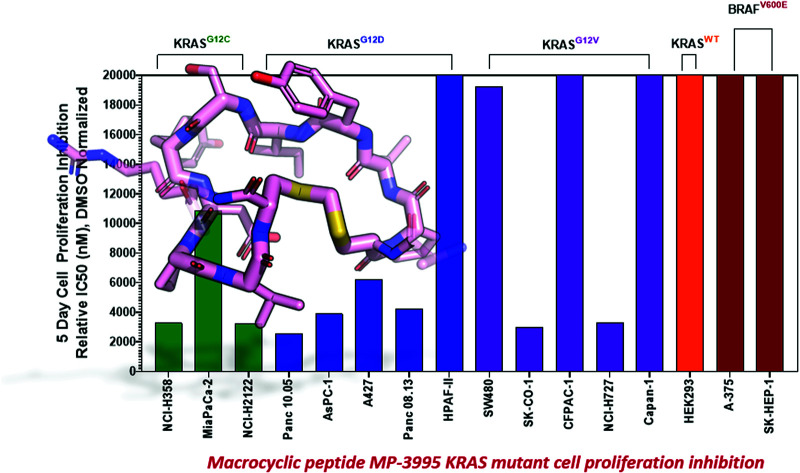

## Introduction

The RAS GTPase serves as a molecular switch to activate signaling cascades related to cell survival and proliferation, most notably, the MAPK and AKT pathways. Human malignancies gain growth advantages by mutating RAS at positions G12, G13, or Q61 to bias the protein to the signaling-active GTP-loaded state.^1^ Indeed, RAS is the most mutated oncogene across human cancers. Amongst the different isoforms, KRAS is the most frequently mutated and is especially prevalent in pancreatic, lung, and colorectal cancers.^1^ Although mutant KRAS was discovered to be a common driver of human cancers in the early 1980s, there were, until very recently, no approved therapeutics against this target. Fortunately, recent advances have reinvigorated the field. Small molecule covalent inhibitors of KRAS^G12C^ have shown efficacy in animal models and in the clinic.^2,3^ One of these, sotorasib (AMG 510, LUMAKRAS™), was recently approved for treatment in patients with KRAS^G12C^ driven non-small cell lung cancers that are either metastatic or locally advanced.^4^ Despite these remarkable advances, significant challenges remain for targeting tumors driven by KRAS with non-G12C mutations. Specifically, the current clinical molecules rely on a covalent modifier strategy that has strict specificity for the C12 residue, which is present in humans at a prevalence of only 3.4% in colorectal cancers and in 7.4% of non-small-cell lung cancer (NSCLC) cases.^5^ Furthermore, as clinical studies progress with G12C inhibitors, resistance mechanisms to these new drugs are being mapped, including identification of additional changes to the KRAS protein itself.^6^ For the remaining more prevalent KRAS mutations (*e.g.*, G12D, G12V), significant challenges remain. Specifically, campaigns to identify small molecule binders to KRAS have largely failed due to a paucity of surface pockets suitable for small molecule docking. One available druggable pocket appears to be the nucleotide binding site, but it is normally occupied by GDP or GTP, which have millimolar cellular concentrations and picomolar affinities for KRAS, hence posing a major challenge to the development of competitive inhibitors. These challenges have prompted investigators to consider alternative approaches. Amongst these, peptide-based modulators hold promise due to their propensity to bind to diverse protein epitopes and modulate their activity. Advances in display-based technologies^7,8^ have led to successful screens on multiple protein targets resulting in novel peptide hits, increasing the promise of this emerging modality as a complementary way to modulate protein function.

**Fig. 1:**
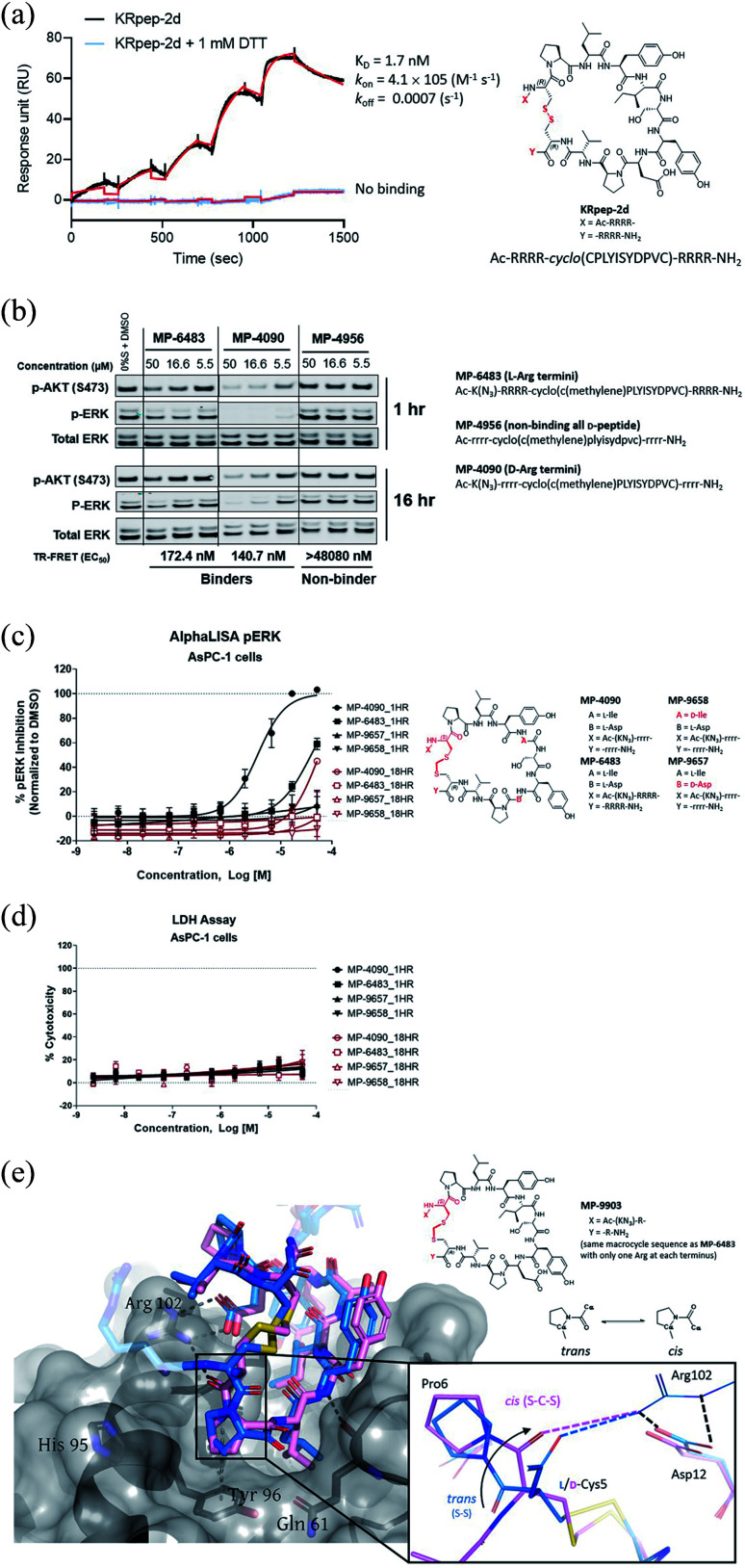
Replacement of the KRpep-2d's disulfide bridge with a d-Cys^5^–CH_2_–l-Cys^15^ thioacetal linkage results in a redox-stable, high affinity peptide. (A) SPR analysis shows that oxidized (cyclic) but not reduced (linear) KRpep-2d binds with high affinity to GDP-loaded KRAS^G12D^. The chemical structure is shown, with the N- and C-terminal arginine chains and disulfide linker highlighted in red. (B) Redox-stable peptides MP-6483 and MP-4090 display cell-based inhibition of KRAS signaling (pERK and pAKT) in AsPC-1 cells, as assessed by Western blot at 1 hour and 16 hour time-points; non-binding control enantiomeric peptide MP-4956 showed no activity (Left). Sequences for the peptides are shown. (C) MP-6483 and MP-4090 but not the non-binder controls (MP-9657 and MP-9658) display cell-based inhibition (pERK) in AsPC-1 cells, as assessed by Alpha SureFire Ultra Multiplex Phospho/Total ERK1/2 assay (Perkin Elmer) when treated for 1 h (*n* = 6, black symbols) and 18 h (*n* = 2, red symbols). The d-Cys^5^ residue, N- and C-terminal arginine residues, core modifications and thioacetal linker is highlighted in red (D) the same lysates in panel C were also assessed for membrane toxicity, as measured by the CytoTox-ONE™ homogenous membrane integrity assay (Promega). (E) Superimposition of co-crystal structures involving (i) KRpep-2d in complex with KRAS^G12D^ (GDP) (PDB ID 5XCO, blue) and (ii) MP-9903, a peptide containing the d-Cys^5^–CH_2_–l-Cys^15^ thioacetal linkage, with KRAS^G12D^ (GMPPCP) (PDB ID 7ROV, pink), shows a highly similar KRAS conformation and that binding of MP-9903 involves a *cis* peptide bond between d-Cys^5^ and Pro^6^. For clarity, terminal arginine residues are either transparent or hidden. The d-Cys^5^ residue, N- and C-termini and thioacetal linker are highlighted in red in the structure and the termini changes to MP-6483 is noted.

Several research groups have indeed reported high affinity *bona fide* peptide binders to KRAS that represent valuable starting points for drug discovery.^9–12^ In particular, Sakamoto *et al.* used phage display to identify a disulfide cyclized peptide with the sequence Ac-Arg^1^-Arg^2^-Arg^3^-Arg^4^-Cys^5^-Pro^6^-Leu^7^-Tyr^8^-Ile^9^-Ser^10^-Tyr^11^-Asp^12^-Pro^13^-Val^14^-Cys^15^-Arg^16^-Arg^17^-Arg^18^-Arg^19^-NH_2_.^10^ This molecule, termed KRpep-2d (see Fig. 1A), bound KRAS^G12D^ with nanomolar affinity in both the GDP and GTP analog loaded states. Alanine-scanning mutagenesis identified Leu^7^, Ile^9^, and Asp^12^ as critical binding residues.^13^ X-ray crystallography revealed the binding site to be near Switch II, allosterically blocking the interaction of KRAS with the guanine nucleotide exchange factor, SOS1.^14^ Subsequently we^15^ and others^12^ verified this peptide as having high-affinity and stoichiometric binding to KRAS. Although KRpep-2d represents a promising and novel KRAS binder, we concluded that structural modifications were required to render it cell-active. In particular, the disulfide crosslink is not expected to remain intact within the reducing environment of the cytosol. Furthermore, additional medicinal chemistry optimization could address potential peptide stability and permeability deficiencies.

To achieve cell permeability, cationic and hydrophobic residues are often incorporated into peptides targeting intracellular proteins. However, these design features can confound the interpretation of biochemical and functional assays.^15^ Indeed, some recently reported putative peptidic KRAS inhibitors incorporating these elements were, in fact, false positives.^15^ Thus, we took extra caution in our studies with the KRpep-2d scaffold since it contains several hydrophobic residues and a total of eight exocyclic arginines. In particular, we applied rigorous controls in the experiments described herein to ensure that the macrocyclic peptides we designed were not only *bona fide* KRAS binders but also authentic, on-target cell active molecules. These peptides therefore represent a new approach towards blocking KRAS-driven signaling beyond the G12C mutation, an area of high unmet need.

Using KRpep-2d as the starting point, we sought to improve binding affinity, increase proteolytic stability, and impart membrane permeability to advance a molecule capable of blocking cellular KRAS signaling. After exploring different strategies, we successfully replaced the disulfide bond with a thioacetal crosslink involving a d-Cys residue at position 5. X-ray crystallography revealed that peptides using this macrocyclization motif bind to KRAS in a similar manner but with a *cis* peptide bond between d-Cys^5^ and Pro^6^. Replacing the N- and C-terminal arginine residues with their d-amino acid counterparts produced a peptide with weak cellular activity. Introduction of an α-methyl group at Ser^10^ resulted in a molecule (MP-3995) with prolonged proteolytic stability and cellular blockade of pERK activity in KRAS^G12D^ (AsPC-1) cells but was inactive against a KRAS^WT^ cancer line (A375) harboring BRAF^V600E^, a MAPK pathway activating mutation that is downstream of KRAS. On-target cellular activity was further verified using non-binding peptide controls, counter-screens, and target engagement assays. In a panel of cancer cell lines, MP-3995 inhibited proliferation in KRAS dependent lines but not in KRAS independent lines. Despite these favorable attributes, we identified the arginine-rich nature of this series to be a barrier for further development as strong histamine release was observed in an *ex vivo* assay. Initial attempts to reduce the flanking Arg residues resulted in a loss of membrane permeability and cellular activity. Nevertheless, the peptides described in this report represent a valuable scaffold for the genesis of novel validated inhibitors of KRAS signaling in cancer patients unserved by current successes with covalent G12C inhibition.

## Results

### Replacement of the KRpep-2d disulfide bridge with a d-Cys^5^–Cys^15^ thioacetal linkage results in a redox-stable, high affinity peptide

Sakamoto *et al.*^10^ used phage display to identify the first example of a macrocyclic peptide with *bona fide* high-affinity binding to KRAS, KRpep-2d. The specific binding of this peptide to KRAS^G12D^ was previously independently validated by us using a suite of biophysical approaches including isothermal titration calorimetry (ITC), surface plasmon resonance (SPR), thermal shift assay (TSA), and hydrogen-deuterium exchange mass spectrometry (HDX-MS).^15^ Although this molecule provides an excellent starting point for medicinal chemistry efforts, it contains a disulfide linkage between Cys^5^ and Cys^15^, which might not remain intact in the reducing cytosolic environment and thus lose binding. Indeed, although SPR analysis confirmed high affinity binding (1.7 nM) for KRAS^G12D^ with the disulfide intact, binding was completely lost in the presence of DTT, a result presumably due to disulfide reduction (Fig. 1A). Furthermore, a linear derivative of KRpep-2d where the two cysteines were replaced by l-serine, a cyclization incompetent isostere, exhibited no binding (data not shown). For KRpep-2d, redox sensitivity likely also contributed to the lack of cell-based inhibition of ERK phosphorylation (pERK) in our hands, as measured by western blot (Fig. S1†). Accordingly, we sought to identify a peptide that maintained KRAS binding affinity but with a redox insensitive cross-link. Using a template with an N-terminal 6-azido-l-lysine residue, to enable cellular permeability measurements with the NanoClick assay,^16^ we explored a series of disulfide bridge replacements (Table 1). To assess the peptide binding affinities in high-throughput format, we used a TR-FRET assay employing GDP-loaded KRAS^G12D^ and a FAM-labeled KRpep-2d family member tracer peptide (see ESI†). This assay reports relative binding values whose EC_50_'s are right-shifted compared to the corresponding binding constants (*K*_D_'s) due to the high concentration of tracer peptide used. When the disulfide was replaced with a lactam moiety linking l-Asp at position 5 and l-Dap ((*S*)-2,3-diaminopropionic acid) at position 15, peptide 1, no binding was detected. Intriguingly, the corresponding d-Asp^5^/l-Dap^15^ analog (2) exhibited modest biochemical activity (EC_50_ = 19 331 nM) suggesting a potential benefit from incorporating a d-amino acid at position 5. Next, we explored thioacetal cross-links to connect the cysteine thiols. The l-Cys^5^/l-Cys^15^ thioacetal peptide (3) lost activity when compared to the parent disulfide (TR-FRET EC_50_ = 13 740 nM), similar to previous findings.^13^ Encouragingly, the d-Cys^5^/l-Cys^15^ thioacetal (MP-6483) had much improved biochemical potency (172 nM), a 11-fold improvement over KRpep-2d (Table 1). However, adding another atom to the linker in the d-Cys^5^/l-homoCys^15^ analog (4) led to a marked reduction in TR-FRET EC_50_ (4719 nM). Importantly, the most active peptide from this set of analogs, MP-6483, maintained its binding capacity in both oxidizing and reducing environments (Table 1, Fig. S2†), as assessed by TR-FRET. In agreement with the SPR results, KRpep-2d lost the capacity to bind to GDP-loaded KRAS^G12D^ in the presence of 1 mM DTT (Table 1, Fig. S2†). MP-6483 also showed a weak capacity to block pERK and pAKT signaling downstream of KRAS in the cellular context (Fig. 1B and C), with an absence of membrane disruption, as assessed by a LDH release assay (Fig. 1D). However, MP-6483's potency appeared to weaken significantly over time in AsPC-1 cells (a KRAS^G12D^ pancreatic cancer cell line), especially when assessed by the alphaLISA assay (Fig. 1C), suggesting potential proteolytic stability issues.

**Table 1:** Modification of the disulfide cyclization motif of KRpep-2d, Ac-RRRR-cyclo(CPLYISYDPVC)-RRRR-NH_2_

Compound	Sequence	Linker	KRAS^G12D^ GDP TR-FRET EC_50_ (nM)
KRpep-2d	Ac-RRRR-cyclo(CPLYISYDPVC)-NH_2_	l-Cys^5^–l-Cys^15^	1916 (>50 000^a^)
1	Ac-K(N_3_)-RRRR-cyclo(DPLYISYDPV-Dap)-RRRR-NH_2_	l-Asp^5^–l-Dap^15^ (lactam)	>50 000
2	Ac-K(N_3_)-RRRR-cyclo(dPLYISYDPV-Dap)-RRRR-NH_2_	d-Asp^5^–l-Dap^15^ (lactam)	19 331
3	Ac-K(N_3_)-RRRR-cyclo(C(methylene)PLYISYDPVC)-RRRR-NH_2_	l-Cys^5^–CH_2_–l-Cys^15^ (thioacetal)	13 740
MP-6483	Ac-K(N_3_)-RRRR-cyclo(c(methylene)PLYISYDPVC)-RRRR- NH_2_	d-Cys^5^–CH_2_–l-Cys^15^ (thioacetal)	172 (93^a^)
4	Ac-K(N_3_)-RR-cyclo(c(methylene)PLYISYDPV-hC)-RR-NH_2_	d-Cys^5^–CH_2_–l-homoCys^15^ (thioacetal)	4719

a Reducing conditions (1 mM DTT), see Fig. S2. Lower case letters represent d amino acids.

### X-ray crystallography reveals a *cis* peptide bond conformation at d-Cys^5^–Pro^6^

Having replaced the disulfide crosslink with a redox stable thioacetal moiety that maintained high-affinity binding, we next sought to understand the preference for the d configuration at position 5. Accordingly, we solved the co-crystal structure of KRAS loaded with a non-hydrolysable GTP analog (GMPPCP) bound to MP-9903, a peptide containing MP-6483's same macrocyclic sequence but with only one arginine at the N- and C-termini. The chemical structure of MP-9903 is shown in Fig. 1E. KRpep-2d's structure was reported earlier in complex with KRAS GDP (PDB-ID 5XCO). Both structures overlap with a rms deviation of 0.51 Å excluding the switch I region, which undergoes conformational changes due to different crystal contacts (Fig. 1E). Despite GMPPCP loading of KRAS in the MP-9903 co-crystal structure, the protein adopts the off-state (GDP-loaded) conformation, and the peptide binding pockets can easily be superimposed. Interestingly, the MP-9903 peptide bond connecting d-Cys^5^ and Pro^6^ adopts a *cis* conformation allowing the cyclic part of the peptide to occupy the same binding site. Due to the opposite chirality at residue 5, the N-terminus has a more solvent exposed trajectory. Consequently, the hydrogen bond between the backbone carbonyl oxygen (O) of Arg^4^ and side chain nitrogen of KRAS/Arg^102^Nη is disrupted and Arg^102^ hydrogen bonds to the backbone carbonyl oxygen (O) of d-Cys^5^ due to the amide bond flip. All the other interactions observed between KRAS and KRpep-2d were also observed in the crystal structure of the KRAS – MP-9903 complex (except the interactions that involve the terminal arginine) (Fig. 1E). These interactions were also preserved during molecular dynamics simulations (Fig. S3†). The extension of the linker between d/l-Cys^5^ and Cys^15^ by a methylene (thioacetal) group increases the Cα–Cα distance by 0.65 Å and remains solvent exposed. Similarly, the C-terminus of MP-9903 is solvent exposed and lacks any interactions with KRAS. It matches the KRpep-2d conformation remarkably well (Fig. 1E).

### Ala-scanning mutagenesis identifies key binding residues on the macrocycle

With structural information in hand, we next sought to systematically resolve the contributions to the KRAS binding energy by each residue within the macrocycle. Accordingly, we made a library of singular alanine substitutions focusing on the macrocyclic amino acids of MP-1687, the tetra-arginine peptide analog of MP-6483. The resulting nine alanine mutants were tested for binding in the TR-FRET assay and the data and chemical structure of the parent macrocycle are shown in Table 2. Four alanine mutants (Pro6Ala 5, Leu7Ala 6, Ile9Ala 8, and Asp12Ala 11) caused a significant impairment of binding. Apart from the effect at the Pro^6^ position, these observations agree with those reported for the disulfide-bridged parent KRpep-2d, thus confirming the critical binding roles of Leu^7^, Ile^9^, and Asp^12^. Indeed, these results can be rationalized as the side chains of Leu^7^ and Ile^9^ are buried in the hydrophobic pocket on the surface of KRAS (Fig. S3†) and the side chain of Asp^12^ is involved in a salt bridge with the side chain of Arg^102^ from KRAS. Previous results in the context of KRpep-2d showed that Pro6Ala had only a modest (10-fold) reduction in affinity.^13^ On the other hand, in the context of the extended thioacetal cross-link and inverted stereocenter at position 5, replacement of the constrained proline with an acyclic amino acid (Ala or *N*-Me-Ala) or modification of the ring size (azetidine-2-carboxylic acid (Aze) or pipecolic acid (Pip)) dramatically affected the bound conformation, thus resulting in dramatic loss of affinity (data not shown). This observation is in accordance with the *trans–cis* proline isomerization observed in the co-crystal structure and further suggested that local stabilization of the *cis* amide bond conformation might improve affinity. The side chains of residues Ser^10^ and Tyr^11^ interact with the side chain and backbone of KRAS residue Asp^69^ (Fig. S3†), thus rationalizing the moderate loss in affinity when these residues were substituted with alanine. Binding affinities for three other Ala mutants (*i.e.*, Tyr8Ala 7, Pro13Ala 12, and Val14Ala 13) were perturbed to lesser extent as the side chains of these residues are not involved in any intra or inter KRAS – peptide interactions (Fig. S3†). An *in silico* alanine scan was carried out using the crystal structure with standard protocols,^17^ and the results were found to be in reasonable accordance with experimental data; the smallest destabilization of +0.5 kcal/mol was seen for Val14Ala, while for the destabilized mutants (Pro6Ala 5, Leu7Ala 6, Ile9Ala 8, and Asp12Ala 11) the calculations yielded values from 4–9 kcal/mol (Table S1†).

**Table 2:** Alanine scan on peptide Ac-K(N_3_)-RR-cyclo(c(methylene)PLYISYDPVC)-RR-NH_2_ (MP-1687)^a^

Compound	Modification	KRAS^G12D^ GDP TR-FRET EC_50_ (nM)
MP-1687	None	60
5	Pro6Ala	>48 080
6	Leu7Ala	>48 080
7	Tyr8Ala	691
8	Ile9Ala	>48 080
9	Ser10Ala	1072
10	Tyr11Ala	2430
11	Asp12Ala	>48 080
12	Pro13Ala	427
13	Val14Ala	161
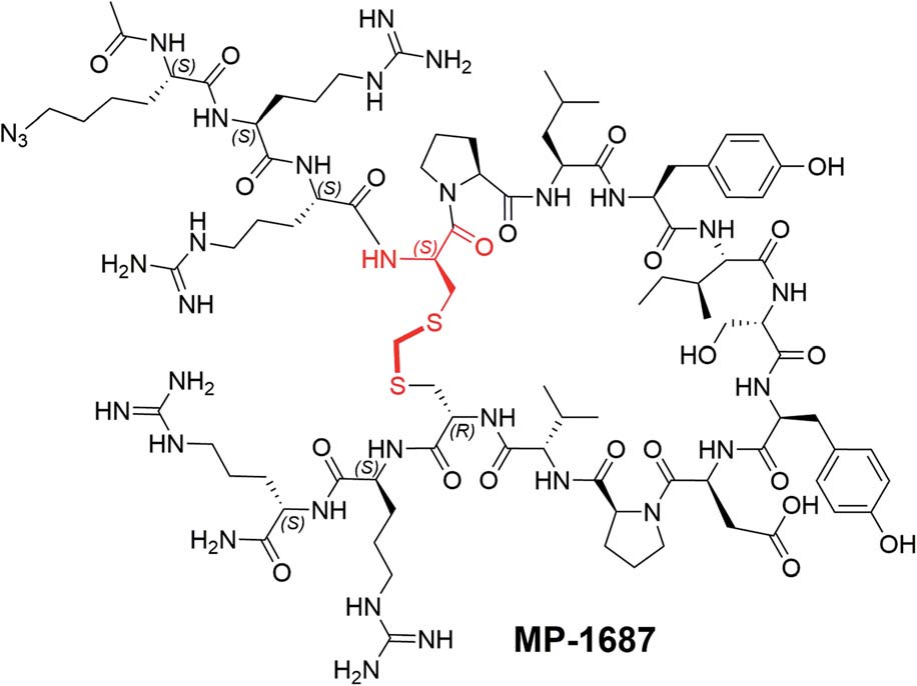		

a The d-Cys^5^ residue and thioacetal linker is highlighted in red in the structure. The N- and C-termini are shown. MP-1687 has the same macrocyclic core as MP-6483, with only two arginine residues at each terminus.

### Replacing the terminal l-Arg residues with d-Arg results in a membrane permeable peptide with improved intracellular stability and on-target cellular activity

**Fig. 2:**
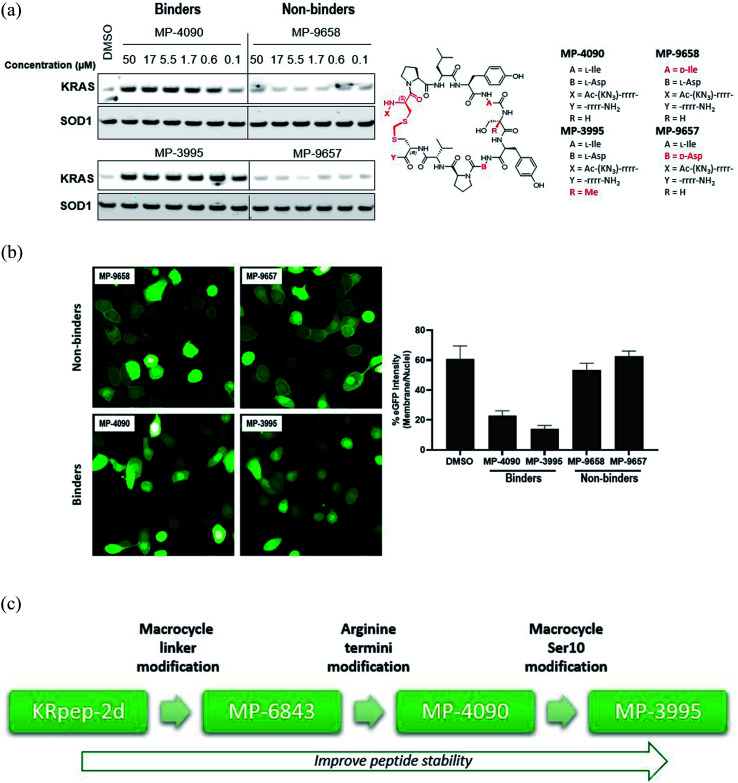
MP-4090 and MP-3995 specifically engage with cellular KRAS to inhibit its interactions with RAF–RBD. (A) Isothermal CETSA® experiments using intact AsPC-1 cells show that both MP-4090 and MP-3995 thermally stabilize KRAS^G12D^ whereas non-binding controls (MP-9657 and MP-9658) do not. The d-Cys^5^ residue, N- and C-termini, core modifications and thioacetal linker are highlighted in red in the structures. (B) Introduction of a RBD-CRD-eGFP fusion protein into AsPC-1 cells (homozygous KRAS^G12D^) using mRNA transfection resulted in enrichment of the GFP signal at the membrane when cells were incubated with DMSO (not shown) or the non-binder control peptides, MP-9657 and MP-9658. Membrane enrichment was lost when cells were incubated with MP-4090 or MP-3995, suggesting that these peptides could effectively compete with the PPI. (C) Summary of SAR evolution from KRpep-2d to MP-3995.

After identifying a binding competent, redox-stable thioacetal linkage, we then sought to improve peptide bioactivity by identifying metabolic soft spots and designing analogs with improved stability. Metabolite identification (MetID) studies on KRpep-2d were performed after incubation in homogenized THP-1 cells and analysis *via* high resolution mass spectrometry. Rapid metabolism was observed, and several metabolites were detected within 10 min that were a result of sequential loss of arginines from one or both termini, as evidenced by changes in mass to charge ratios. Ring opening resulting from disulfide bridge cleavage was not detected, likely due to the non-reducing conditions in the homogenized cells. Accordingly, we decided to replace all eight l-arginine residues on MP-6483 with their hyper-stable enantiomeric counterparts (d-Arg) (Fig. 1B). The resulting peptide, MP-4090, showed permeability as measured by the NanoClick assay (4 hour EC_50_ = 251 nM, 18 hour EC_50_ = 35 nM, Table 3), a result mirrored in imaging studies using FAM-labeled counterparts (Fig. S4†). Importantly, MP-4090 also showed, for the first time, robust inhibition of pERK and pAKT in AsPC-1 cells (Fig. 1B and C). The key binder and non-binder peptide controls described in this manuscript are listed together in Table 3 and the SAR evolution from KRpep-2d is depicted in Fig. 2C.

**Table 3:** Key binder peptides showing SAR evolution and non-binder control peptides

Compound	Peptide sequence^a^	Changes	KRAS^G12D^ GDP TR-FRET EC_50_ (nM)	AsPC-1 pERK EC_50_ 1 h/18 h (μM)	AsPC-1 LDH EC_50_ 1 h/18 h (μM)	A375 pERK EC_50_ 1 h/18 h (μM)	NanoClick EC_50_ 4 h/18 h (nM)
MP-6483 (binder)	Ac-K(N_3_)-RRRR-cyclo(c(methylene)PLYISYDPVC)-RRRR-NH_2_	None	172.4	15.9/>50	>50/>50	NT	2693/636
MP-4090 (binder)	Ac-K(N_3_)-rrrr-cyclo(c(methylene)PLYISYDPVC)-rrrr-NH_2_	All d-Arg	140.7	3.6/30.5	>50/>50	>50/>50	250.5/34.9
MP-3995 (binder)	Ac-KN_3_)-rrrr-cyclo(c(methylene)PLYI-αMeS-YDPVC)-rrrr-NH_2_	All d-Arg, Ser10α-Me-Ser	172.1	1.2/1.5	>50/>50	>50/>50	1777/32.1
MP-4956 (non-binder)	Ac-rrrr-cyclo(c(methylene)plyisydpvc)-rrrr-NH_2_	All d-peptide	>48 080	>50/>50	>50/>50	NT/>50	NT
MP-9657 (non-binder)	Ac-K(N_3_)-rrrr-cyclo(c(methylene)PLYiSYDPVC)-rrrr-NH_2_	All d-Arg, Ile9d-Ile	>48 080	>50/>50	>50/>50	NT	1756/36.2
MP-9658 (non-binder)	Ac-K(N_3_)-rrrr-cyclo(c(methylene)PLYISYdPVC)-rrrr-NH_2_	All d-Arg, Asp12d-Asp	>48 080	>50/>50	>50/>50	NT/>50	196.1/31.4

a Lower case letters represent d-amino acids. NT = not tested.

We also designed a series of non-binding control peptides including an all-d version of MP-6483 (MP-4956) and peptides with enantiomeric substitutions at critical binding residues, Ile^9^ to d-Ile (MP-9657) and Asp^12^ to d-Asp (MP-9658) (Table 3). None of these peptides showed any binding in the TR-FRET assay (EC_50_ > 48 080 nM) nor a capacity to inhibit cellular function (Fig. 1B, C), despite evidence of permeability in the NanoClick assay (Table 3). They also did not induce membrane damage as assessed by the LDH release assay (Fig. 1D). Furthermore, MP-4090 and its non-binding control (MP-9658) were inactive in A375 and SK-MEL-28 cells (Fig. S5A†), counter-screen cell lines harboring BRAF^V600E^, a mutant that activates the MAPK pathway downstream of KRAS. AZ628 (BRAF inhibitor,^18^) and U0126 (MEK inhibitor,^19^), small molecules that inhibit the pathways downstream of KRAS, inhibited MAPK signaling in these BRAF mutant lines in the expected manner (Fig. S5A†). Live-cell imaging using FAM-labelled versions of these macrocyclic peptides confirmed that permeability was comparable between AsPC-1 and A375, but poorer in SK-MEL-28 (Fig. S5B†). In addition, MP-4090 had no effect on upstream EGF receptor activation (Fig. S6A†) and TNFα-stimulated NFκB signaling (a KRAS-independent pathway) (Fig. S6B†) in AsPC-1 cells. This series of control experiments suggested that the cellular KRAS-inhibitory activity measured for MP-4090*via* inhibition of ERK phosphorylation was indeed on-target.

### Stabilizing the Tyr^8^/Ile^9^ metabolic soft spot gives a peptide with sustained cellular activity

Having validated MP-4090 as a cell permeable peptide with on-target pERK inhibition, we next sought to improve its observed cellular potency. Although the exocyclic arginines had been replaced by their d-enantiomers, it appeared that the peptide was still susceptible to proteolytic cleavage as observed by the loss of pERK activity at 18 hours *vs.* 1 hour (Fig. 1C). Indeed, MetID studies on the KRpep-2d scaffold without the terminal arginines performed in homogenized THP-1 cells had also revealed an additional proteolytic soft spot between Tyr^8^ and Ile^9^ of the macrocycle.

To stabilize the peptide against proteolysis, we synthesized analogs with Tyr^8^ and Ile^9^ modifications including α-methylation and backbone homologation, however all were inactive in the TR-FRET assay (data not shown). We then expanded the α-methyl scan to additional residues and tested the resulting peptides for binding and stability (Table 4). Due to challenges with detecting highly cationic 8-Arg peptides on MS instrumentation, we carried out this study on the tetra-arginine parent peptide MP-1687, described in Table 2 previously. α-Methylation was tolerated at positions 10 (Ser, 17) and 13 (Pro, 20) without any change in binding affinity when compared to MP-1687, with a modest 4-fold loss observed at position 14 (Val, 21). In contrast, potency loss was observed at all other positions, ranging from 30-fold at Tyr^11^ to 800-fold at positions 8 or 15. Position 5 and 9 were not investigated. Comparison of the HeLa cell homogenate half-lives of the α-methylated analogs to the parent MP-1687 showed improvements ranging from >10-fold for position 8 to >4-fold for position 10 and >3-fold for position 13 modifications. Considering both the potency and stability data, the α-methyl-Ser^10^ mutation was selected for inclusion in future designs. Incorporating the α-methyl-Ser^10^ modification into our previous lead sequence (MP-4090) led to MP-3995, a peptide with sustained pERK inhibition with EC_50_ values of 1.2 and 1.5 μM at 1 and 18 hours, respectively (Table 3), eliminating the 8-fold cell potency loss seen with MP-4090 at 18 h *vs.* 1 h. This peptide also did not induce membrane damage as assessed by the LDH release assay and did not inhibit pathway signaling in the RAS-independent A375 cell line with the BRAF^V600E^ mutation (Table 3). The key steps in SAR evolution from KRpep-2d to MP-3995 is depicted in Fig. 2C.

**Table 4:** α-Methyl amino acid scan biochemical assay and cell homogenate half-life data on peptide Ac-K(N_3_)-RR-cyclo(c(methylene)PLYISYDPVC)-RR-NH_2_ (MP-1687)

Compound	Modification	KRAS^G12D^ GDP TR-FRET EC_50_ (nM)	HeLa *t*_1/2_ (min)
MP-1687	None	60	37
14	α-Me-Pro^6^	10 830	142
15	α-Me-Leu^7^	11 510	114
16	α-Me-Phe^8^	46 120	>373
17	α-Me-Ser^10^	62	154
18	α-Me-Tyr^11^	1800	71
19	α-Me-Asp^12^	3705	41
20	α-Me-Pro^13^	67	134
21	α-Me-Val^14^	222	NA
22	α-Me-Cys^15^	48 080	120

### MP-4090 and MP-3995 specifically engage with cellular KRAS to inhibit its interactions with RAF–RBD

Since macrocyclic peptides aimed at intracellular targets are prone to cell-based false positives (*e.g.* ref. 15), we sought further evidence of the on-target nature of the current peptide series. Accordingly, we showed that MP-4090 and MP-3995, but not two non-binding controls (d-Ile^9^ analog MP-9657 and d-Asp^12^ analog MP-9658), specifically engage and thermally stabilize KRAS in an isothermal cellular thermal shift assay (CETSA®) using intact AsPC-1 cells (Fig. 2A). The CETSA study was performed under a license from Pelago Biosciences. Target engagement was also supported by the capacity of MP-4090 and MP-3995 to inhibit the interaction of KRAS with an engineered binding reporter; a RAF RBD-CRD-eGFP fusion protein that was introduced into AsPC-1 cells using mRNA transfection. In the presence of DMSO or incubation with non-binder peptides MP-9658 or MP-9657, there was a distinct accumulation of the eGFP fluorescence at the cell membrane, presumably due to its interaction with KRAS. However, when the experiment was repeated with the addition of MP-4090 or MP-3995, this staining pattern was lost and the eGFP signal became diffuse (Fig. 2B), suggesting that the KRAS binding peptides entered the cell, bound to KRAS, and disrupted the interactions with its signaling effectors. Control eGFP fusions whose membrane localization was not dependent on an interaction with KRAS (eGFP with a C-terminal farnesylation signal from HRAS, Fig. S7A;† eGFP with an N-terminal palmitoylation signal from Neuromodulin, Fig. S7B†) showed membrane accumulations with both binder and non-binder peptides.

### Exploring the mechanism of KRAS inhibition

**Fig. 3:**
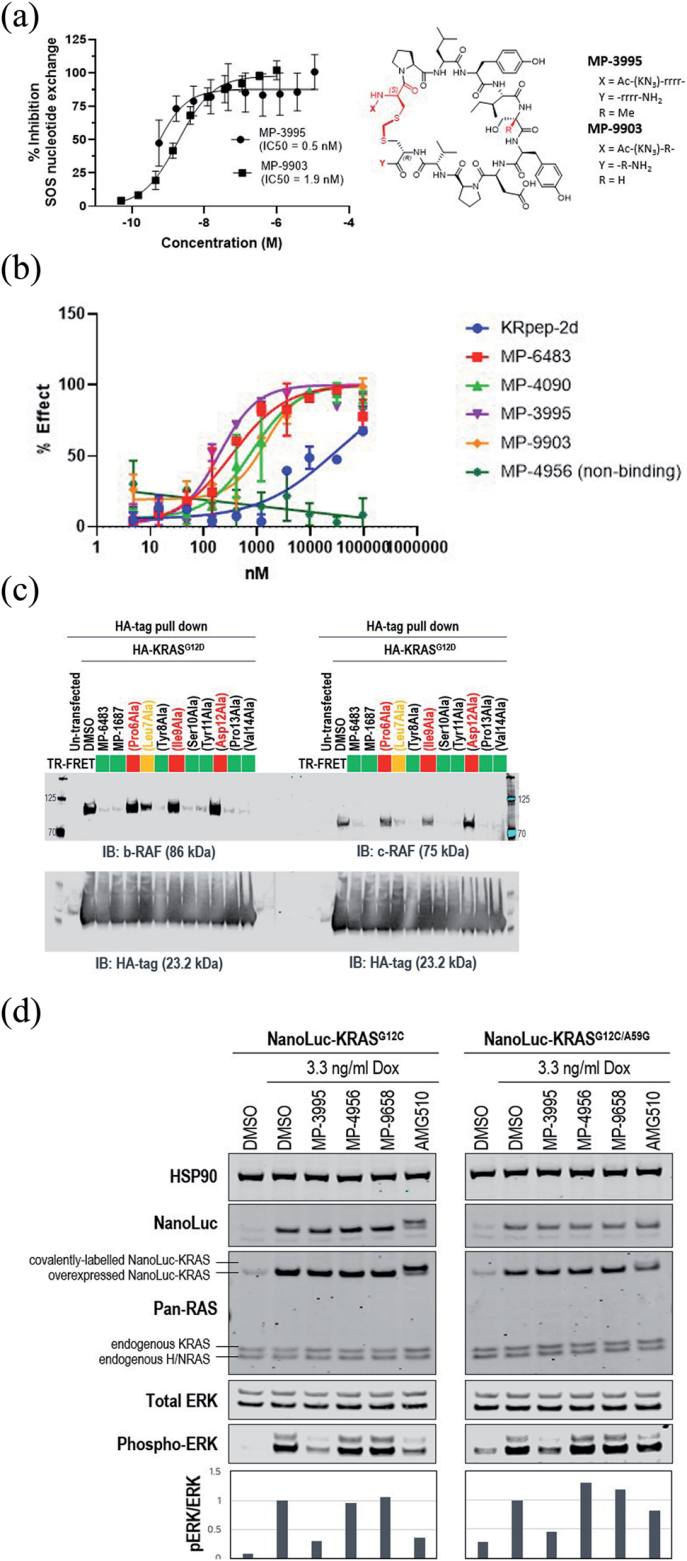
KRpep-2d peptide analogs have dual inhibitory mechanisms. (A) MP-3995 and MP-9903 potently inhibited SOS-mediated nucleotide exchange. The d-Cys^5^ residue, N- and C-termini, core modifications and thioacetal linker are highlighted in red in the structure. (B) MP-6483, MP-4090, MP-3995, and MP-9903 showed a superior capacity to block the KRAS-RBD PPI compared to KRpep-2d, whereas the non-binder control (MP-4956) had no activity. (C) MP-6483 and MP-1687 blocked the co-immunoprecipitation of b-RAF (left panel) and c-RAF (right panel) with KRAS^G12D^. Alanine mutant peptide library analogs of MP-1687 (Table S1†) demonstrated that KRAS binding affinity correlated well with disruption of the PPI. (D) MP-3995 blocked phospho-ERK signaling in cells expressing either NanoLuc-KRAS^G12C^ or NanoLuc-KRAS^G12C/A59G^ whereas AMG 510 only inhibited NanoLuc-KRAS^G12C^, non-binders (MP-4956 and MP-9658) had no activity.

Understanding the inhibitory mechanism of this peptide series is required to assess its potential as a therapeutic and justify further evaluation. We considered two distinct, non-mutually exclusive, mechanisms of action for this peptide series. First, the peptide might inhibit SOS-mediated nucleotide exchange, thus trapping KRAS in the inactive GDP-loaded state. Alternatively, although the peptide binding site does not overlap with the RAF RAS binding domain (RBD) binding site, the peptide might act as a GTP-state allosteric inhibitor to block the PPI and therefore KRAS signaling. Comparison of the KRAS–MP-9903 (Fig. 1E) complex with the KRAS–SOS (PDB 7KFZ) and KRAS–RBD (PDB 6XHB) complexes suggested that both mechanisms could potentially contribute to the inhibition of KRAS signaling. Specifically, binding of MP-9903 should sterically/directly block the binding of SOS protein (Fig. S8A†). On the other hand, structural considerations suggest these peptides could act as allosteric inhibitors of RBD binding. Specifically, RBD binds at the PPI interface between the switch I and switch II regions, whereas MP-9903 binds at the allosteric pocket between Switch II/helix H2 and helix H3. Upon binding of RBD, H2 moves towards H3 (Fig. S8B†), however binding of MP-9903 moves the switch II and helix H2 towards the PPI interface. Such an outward conformation of switch II and helix H2 is not compatible for the binding of RBD, thus allosterically blocking RBD binding and KRAS signaling (Fig. S8C, D†). In agreement with this analysis and previous biochemical findings for KRpep-2d,^10,12,13^ our improved analogs blocked both GDP- and GTP-state activities of KRAS. Specifically, MP-3995 and MP-9903 potently inhibited SOS mediated nucleotide exchange (Fig. 3A). Compared to KRpep-2d, our improved analogs (MP-6483, MP-4090, MP-3995, and MP-9903) were also superior at inhibiting the interaction between KRAS^G12D^ and GST-RBD in a biochemical PPI assay (Fig. 3B). We also probed PPI disruption with full-length, cellular KRAS, showing that MP-6483 was highly effective at inhibiting the pulldown of endogenous b-RAF and c-RAF with HA-tagged KRAS^G12D^ from cell lysates (Fig. 3C). Furthermore, studies with our alanine scan panel peptides (Table 2) demonstrated that PPI disruption efficiency corelated well with their TR-FRET binding affinities as effective blockade of RAF pull-downs was seen with binder peptides, with intermediate effects with the Leu7Ala analog, a peptide with moderate affinity. No effect was seen with non-binder peptides from the panel, the alanine mutants of Pro^6^, Ile^9^ or Asp^12^. The capacity to directly inhibit the GTP-loaded state was further supported by the lack of potency shift in our pERK assay when AsPC-1 or NCI-H358 (KRAS^G12C^) cells were treated with EGF (Fig. S9A and B†), a stimulus that shifts the nucleotide-loaded state towards GTP-bound KRAS. In contrast, the expected potency shift was observed with a GDP-state-selective G12C inhibitor (MRTX-1257)^3^ in the presence of EGF treatment (Fig. S9B†). The capacity of these peptides to directly inhibit the GTP-loaded state was probed using HEK293 cells expressing either NanoLuc-KRAS^G12C^ or NanoLuc-KRAS^G12C/A59G^ under doxycycline control. A59G is a mutation that abrogates the remaining basal level of GTPase activity in KRAS^G12C^, thus pushing the protein more fully into the GTP state.^20^ As expected, induced expression of the G12C and G12C/A59G mutant proteins led to increased KRAS signaling, as measured by pERK levels (Fig. 3D). Treatment with the GDP-state preferring G12C inhibitor AMG 510 (sotorasib) resulted in covalent modification of the NanoLuc-KRAS^G12C^ target protein as evidenced by the increase in molecular weight on the Western blot (Fig. 3D), something not seen with NanoLuc-KRAS^G12C/A59G^. AMG 510 treatment also blocked NanoLuc-KRAS^G12C^ but not Nanoluc-KRAS^G12C/A59G^ signaling, as indicated by pERK levels (Fig. 3D). Together, these observations are consistent with the capacity of AMG 510 to exclusively bind to and inhibit GDP-loaded KRAS^G12C^ and for the A59G mutation to further bias the protein to the GTP state. In contrast, MP-3995 was effective at inhibiting both the single and double mutants (Fig. 3D), suggesting that the peptide can inhibit signaling even when KRAS is pushed more completely into the active conformation.

### MP-3995 inhibits pERK and cell proliferation in a panel of mutant KRAS cell lines

**Fig. 4:**
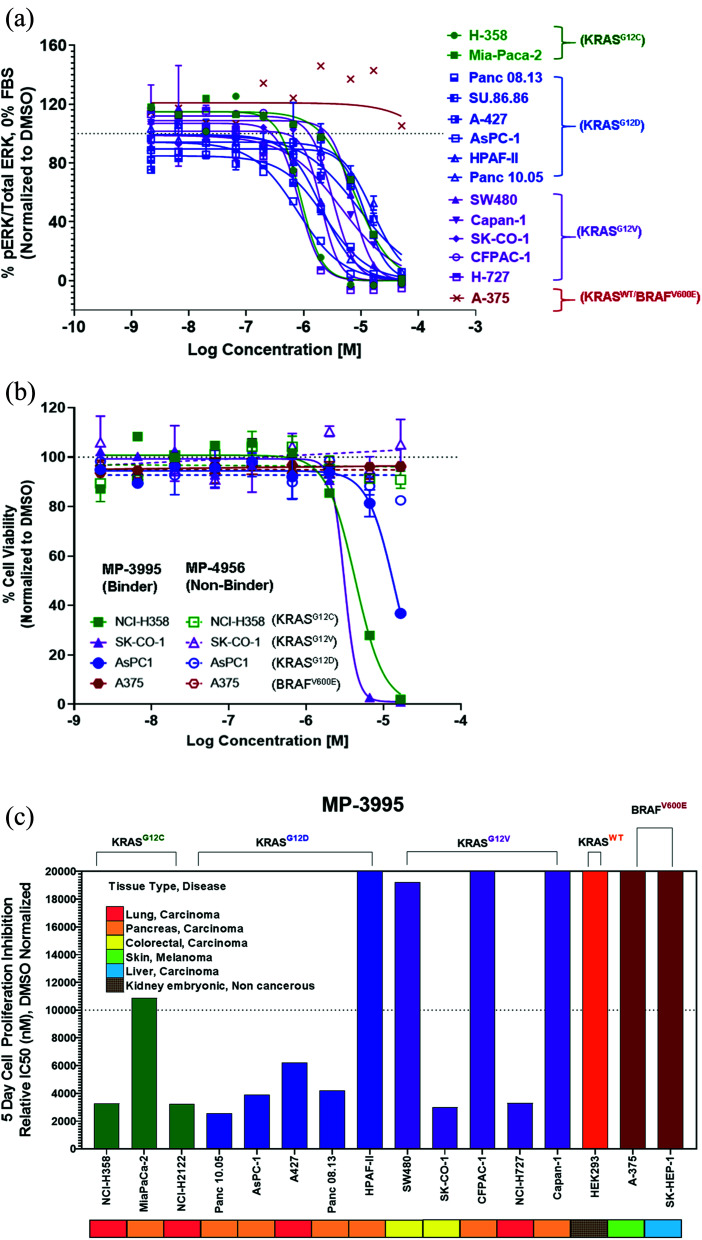
MP-3995 inhibits cell proliferation in cell lines of different tissue origins bearing various KRAS mutations. (A) MP-3995 inhibits phospho-ERK in KRAS mutant cell lines but not BRAF^V600E^ (KRAS independent) A375 cells. (B) MP-3995, but not the non-binder control MP-4956, inhibits cell proliferation in KRAS^G12D^, KRAS^G12C^ and KRAS^G12V^ mutant cells. (C) MP-3995 inhibits cell proliferation in 8 out of 13 KRAS mutant cell lines across different disease indications.

Next, we probed whether the dual inhibitory mechanism of MP-3995 could translate into pERK inhibition across a panel of cells lines – a dataset aimed at understanding the scope of genetic backgrounds this peptide could be applied to. In a variety of KRAS^G12C^, KRAS^G12V^, and KRAS^G12D^ cancer lines, MP-3995 blocked pERK signaling in the low micromolar range (Fig. 4A), thus demonstrating this molecule is a pan-KRAS inhibitor. These effects also translated to inhibitory effects on cell proliferation in a KRAS^G12D^ line (AsPC-1 cells) as well as lines harboring KRAS^G12V^ (SK-CO-1) and KRAS^G12C^ (NCI-H358 and NCI-2122) (Fig. 4B). Importantly, the all-d non-binding control peptide, MP-4956, had minimal inhibitory effects in these lines, suggesting an on-target effect (Fig. 4B). Mostly importantly, MP-3995 had no effect on pERK inhibition in A375 cells (Fig. 4A), consistent with the lack of cell growth inhibition by the peptide in this line and the on-target nature of the molecule (Fig. 4B). In a separate experiment, we identified different cell lines sensitive and insensitive to MP-3995 (Fig. 4C). For the latter, this included HEK293 cells (Fig. 4C), a cell line that has no dependency on RAS for cell proliferation.^21^ Overall, this extended cell proliferation panel showed cell proliferation effects for eight out of thirteen cell lines.

### The induction of mast cell degranulation by arginine-rich KRpep-2d analogues represents a barrier to their progression as therapeutics

Although the datasets collected illustrated on-target KRAS inhibition in the cellular context, we were concerned that the current polybasic nature of these peptides might limit their application in the *in vivo* setting. Indeed, cationic peptides can induce IgE-independent mast cell degranulation (MCD,^22,23^), a process involving the release of biogenic amines (most notably histamine), as well as a cocktail of proteases, cytokines, leukotrienes and prostaglandins.^24^ This can result in itchiness, hives, edema, and even death through anaphylactic shock. Unfortunately, KRpep-2d (data not shown) and analogues, including MP-4090, proved to be highly potent activators of MCD, as determined by an *ex vivo* histamine release assay using rat mast cells. To investigate further, we probed histamine release for a series of MP-4090 peptides containing varying numbers of arginines on the N- and C-termini (Table 5). The reported rat mast cell degranulation (rMCD) value in Table 4 is a threshold for the lowest concentration at which a two-fold change in histamine release was observed. Arginine content correlated with the level of histamine release in these cells, with peptides containing 0–2 arginines (MP-9903, 24 and 25) being free from this potential liability. Interestingly, MCD activity was observed to be inversely correlated to peptide permeability as monitored by the NanoClick assay, leading to a lack of cellular activity when the Arg-count was less than 8. The MCD liability is independent of the stereochemistry of the arginine tails. Of note, the binding affinity was minimally affected by the number and stereochemistry of arginine residues.

**Table 5:** Arginine-truncation and mast cell degranulation SAR study

Compound	Sequence^a^	KRAS^G12D^ GDP TR-FRET EC_50_ (nM)	AsPC-1 pERK EC_50_ 1 h/18 h (μM)	# of Arg	rMCD histamine release threshold (μM)	NanoClick EC_50_ 4 h/18 h (nM)
MP-4090	Ac-K(N_3_)-rrrr-cyclo(c(methylene)PLYISYDPVC)-rrrr-NH_2_	140.7	3.6/30.8	8	≥0.14	251/35
MP-6483	Ac-K(N_3_)-RRRR-cyclo(c(methylene)PLYISYDPVC)-RRRR-NH_2_	172.4	15.9/>50	8	≥0.14	2693/636
23	Ac-K(N_3_)-RRR-cyclo(c(methylene)PLYISYDPVC)-RRR-NH_2_	75.8	>50/>50	6	≥0.14	3314/>10 000
MP-1687	Ac-K(N_3_)-RR-cyclo(c(methylene)PLYISYDPVC)- RR-NH_2_	59.4	>50/>50	4	≥3.7	4552/4711
MP-9903	Ac-K(N_3_)-R-cyclo(c(methylene)PLYISYDPVC)-R-NH_2_	138.6	>50/>50	2	>100	6939/6828
24	Ac-K(N_3_)-cyclo(c(methylene)PLYISYDPVC)-R-NH_2_	187.6	>50/>50	1	>100	>10 000/4462
25	Ac-K(N_3_)-cyclo(c(methylene)PLYISYDPVC)-NH_2_	409.5	>50/>50	0	>100	>10 000/5136

a Lower case letters represent d-amino acids.

## Discussion

The recent landmark approval of sotorasib (LUMAKRAS™) for KRAS^G12C^ mutated non-small cell lung cancer (NSCLC), provides definitive pharmacological validation of this target for human cancers. This long-sought victory will undoubtedly benefit a subset of patients. However, for the large number of cancers driven through non-G12C mutant KRAS (*e.g.*, G12D and G12V), much work remains. For these mutations, leveraging the switch II pocket with non-covalent analogs is an obvious approach. However, it is uncertain whether this strategy will be successful. As well, given the importance of KRAS and its associated challenges, orthogonal approaches need to be pursued.

The discovery of KRpep-2d, a macrocyclic peptide with validated high-affinity binding, represented an attractive starting point for the identification of a non-G12C KRAS inhibitor. However, we recognized that the disulfide-mediated macrocyclization strategy represented a barrier to cellular activity since it would likely be rapidly reduced leading to the non-binding linear form in the intracellular compartment. Indeed, in our hands, this molecule had no effect on cellular KRAS signaling and exhibited a very short half-life in cell homogenate. Most attempts to replace the disulfide bond with a non-reducible linkage resulted in peptides with forfeited KRAS binding. However, by extending our search to include variation of stereochemical centers, we discovered that the linkage of a d-Cys^5^ residue to Cys^15^ through a thioacetal bridge resulted in a redox stable, high affinity binder. Next, replacing the proteolytically unstable N- and C-terminal tetra-arginine tails with their enantiomeric counterparts, resulted in a peptide with cellular activity. Addressing a metabolic soft spot within the macrocycle through a Ser^10^ to α-methyl-Ser substitution further improved metabolic stability and consequently sustained cellular activity (Table 3, Fig. 2C). Such constrained building blocks can force peptides into their biologically active conformations, while often providing remarkable resistance to enzymatic degradation.

Studies by us and others^10–13^ suggest that the KRpep-2d peptides series might inhibit KRAS signaling in at least two distinct ways, by directly blocking the interaction with KRAS effectors (*e.g.*, RAF) as well as by indirectly preventing these interactions by blocking the conversion of the GDP (off) state to the GTP (on) state. Both activities may indeed be contributing to cellular KRAS inhibition. Dual inhibition of mutant KRAS signalling is attractive, especially considering the observation that cancer cells can reactivate the MAPK pathway to resist G12C covalent inhibitors,^25^ molecules that trap the protein in the GDP state.^21^ Initial studies suggest that such compensatory mechanisms may not occur with KRpep-2d family members. First, pathway up-regulation with EGF stimulation did not alter the potency of MP-4090 (Fig. S9†). In addition, biasing KRAS protein to the GTP (on) state with the G12C/A59G double mutant prevented pERK inhibition with AMG 510 (sotorasib) but not MP-3995 (Fig. 3D), suggesting that this peptide can continue to prevent mutant KRAS signaling even when the protein is pushed into the active state.

A key foundation for the identification of *bona fide*, functionally active peptides targeting intracellular proteins is the application of stringent experimental controls, both chemical and biological in nature. This is important as cationic and hydrophobic elements that are often highly prevalent in cell penetrant peptides can lead to false positive cellular read-outs through cell membrane disruption. Indeed, this has been shown specifically for peptides that have been incorrectly reported to have on-target cellular activity against KRAS.^15^ Recently, it was demonstrated that these features can also lead to phospholipidosis, at least when present in small molecules.^26^ Thus, we applied a host of chemical and biological controls in our studies. For the former, we made a series of control peptides designed to be as similar as possible to the cell active molecules but devoid of KRAS binding. This was accomplished through stereo-inversion of all or key binding amino acids. The observation that these peptides were indeed non-binders but also inactive in our pERK assay suggested that the cell activity of our lead peptides (MP-4090 and MP-3995) was due to the consequences of KRAS binding. A series of biological controls gave us further confidence that these peptides had *bona fide* cellular activity. Indeed, the absence of LDH release and off-target activity in an irrelevant signaling pathway was consistent with the on-target nature of this series. As well, the lack of pERK and proliferation inhibition activities in a KRAS independent line (A375) further suggested that off-target mechanisms were not involved. In addition, evidence of direct target engagement was provided by peptide-induced KRAS thermal stabilization in a CETSA® assay and through the capacity of these peptides to displace GFP–RBD–CRD protein from the cell membrane. In both cases, non-binding control peptides gave negative results, as expected. Overall, given the propensity of macrocyclic peptides for both biochemical and cellular false-positive signals,^15^ we advocate that such studies routinely apply an array of biological and chemical controls. A relevant counterexample to our work is that published by Sakamoto *et al.*^11^ This work described an interesting set of amino acid substitutions (including non-natural residues) and disulfide replacements (including bicyclic designs) to the KRpep-2d scaffold. Although the studies were extensive, we found the results difficult to interpret since there was a paucity of both biological and chemical controls. In particular, the peptides were not tested for membrane disruption activity which could artificially influence pERK readouts due to the leakage of cellular contents. In addition, other relevant counter-screens were not applied – such as those involving KRAS independent lines (*e.g.*, HEK293 and A375). The work we reported here highlighted the lack of cell permeability in the original KRpep-2d scaffold which we successfully engineered and validated using cell permeability readouts like imaging and our internally developed NanoClick assay.^16^ It was not clear if KS-58 was cell permeable based on the reported data. In addition, no evidence for cellular target engagement was provided and cell proliferation assays were conducted at a single, relatively high (30 μM), concentration, rather than in a dose dependent manner. The reported *in vivo* study was also particularly challenging to interpret since the xenografts did not grow from day 5 to day 29 in the control group. Despite these shortcomings, KS-58 may indeed represent important improvements to the original peptide and should be considered for future efforts aimed at advancing the KRpep-2d family of peptides towards the clinic.

The reliance on poly-arginine sequences at the N- and C-termini make it challenging to progress the KRpep-2d analogs studied here toward the clinic. Indeed, *ex vivo* mast cell degranulation studies confirmed a strong correlation between the number of arginine residues and histamine release, a phenomenon that can lead to serious consequences *in vivo*. Unfortunately, reducing the number of arginines also correlated with a loss of cell permeability and subsequent cellular activity, therefore confounding their pipeline progression.

Four decades of KRAS research has started to bear fruit with the 2021 approval of the first direct inhibitor of a specific KRAS mutation, G12C. The search for efficacious inhibitors of all other mutant KRAS-driven cancers continues. Despite the structural liabilities identified by us, the peptides described herein represent valuable templates for achieving *in vivo* activity against KRAS-driven, non-G12C cancers. These efforts would focus on identification of sequence variants that achieve cell entry without dependence on arginine-rich sequences. Our efforts directed towards such objectives will be the subject of future communications.

Overall, systematic studies reported here made key advances on this peptide series and used rigorous controls to validate the potential of blocking mutant-KRAS function in cancer cells *via* binding to this unique epitope. As such, novel avenues are open for impacting KRAS-mediated cancers beyond the recent successes with covalent G12C modulation.

## Conflicts of interest

There are no conflicts to declare.

## Data availability

Additional information is available in the ESI† file including supplemental figures, materials and methods and peptide characterization.

## Author contributions

Set research direction, performed experiments, interpreted data, wrote the paper: S. L., N. Boyer, N. Boo, C. H., G. V., R. D., T. Y. Y., S. N. and S. K. Performed experiments, interpreted data: Y-C. A. J., M. G., K. K., K. M. P., A. Sadruddin, P. G., P. O., L. G., X. Y., B. B., F. C. and E. W. Set research strategy, interpreted data: C. J. B., C. V., R. J. G., N. J. L., A. Stoeck, A. P., B. H., T. K. S. and D. P. L. Set research strategy, interpreted data, wrote the paper: C. W. J., K. B. and A. W. P.

## Supplementary Material

SC-012-D1SC05187C-s001
